# Indication for delivery and neonatal outcomes in prenatally diagnosed gastroschisis: A 20‐year retrospective cohort study

**DOI:** 10.1002/pmf2.70333

**Published:** 2026-06-03

**Authors:** Nikan Zargarzadeh, Giulia Bonanni, Enaja Sambatur, Ehsan Rojhani, Eyal Krispin, Kjersti Aagaard, Alireza A. Shamshirsaz

**Affiliations:** ^1^ Fetal Care and Surgery Center (FCSC) Division of Fetal Medicine and Surgery Boston Children's Hospital Harvard Medical School Boston Massachusetts USA; ^2^ Comprehensive Fetal Care Center at Dell Children's, Division of Fetal Medicine and Therapy Department of Women's Health, Dell Medical School The University of Texas Austin Texas USA; ^3^ Obstetrics and Gynecology Department HCA Healthcare and HCA Healthcare Research Institute Nashville Tennessee USA; ^4^ Obstetrics and Gynecology Department HCA Texas Maternal Fetal Medicine Houston Texas USA

**Keywords:** delivery timing, gastroschisis, neonatal outcomes, prenatal diagnosis

## Abstract

**Background:**

The optimal timing and indication for delivery in gastroschisis remain subjects of ongoing debate. This study evaluates clinical characteristics and postnatal outcomes associated with varying indications for delivery in this population.

**Methods:**

This Institutional Review Board approved retrospective cohort study included all pregnancies with a prenatal ultrasound diagnosis of gastroschisis delivered between 34 0⁄7 and 38 6⁄7 weeks’ gestation at a single tertiary care center from January 2000 through December 2023. This gestational age range represents a clinically relevant window encompassing late‐preterm and early‐term births, during which delivery timing is frequently influenced by physician decision‐making rather than spontaneous labor. Pregnancies were categorized according to indication for delivery into three groups: (1) *medically indicated delivery*, defined as delivery prompted by maternal, fetal, or placental conditions, including abnormal fetal surveillance findings (non‐stress test or biophysical profile); (2) *spontaneous labor*, defined as spontaneous onset of labor without preceding fetal surveillance abnormalities or ultrasound‐based concerns; and (3) *fetal bowel concern*, defined as delivery prompted by ultrasound findings suggestive of bowel compromise, including persistent bowel dilation, bowel wall thickening, or increased echogenicity. Neonatal outcomes assessed included age at abdominal wall closure, number of surgical interventions within the first month and first year of life, length of hospital stay, incidence of sepsis, age at initiation of enteral feeding, and rate of complex gastroschisis. Comparisons of outcomes across delivery indication groups were adjusted for gestational age at delivery.

**Results:**

A total of 83 infants met the inclusion criteria, including 22 delivered for fetal bowel concern, 36 for medically indicated delivery, and 25 following spontaneous labor. Age at abdominal wall closure occurred significantly earlier in the medically indicated delivery and spontaneous labor groups compared with the fetal bowel concern group after adjustment for gestational age at delivery (adjusted *p* = 0.013 and *p* = 0.025, respectively). There were no significant differences across delivery indication groups in the number of surgical interventions within the first month or first year of life, length of hospital stay, incidence of sepsis, age at initiation of enteral feeding, or rate of complex gastroschisis.

**Conclusion:**

Although bowel‐related concerns frequently prompted delivery, this approach was not associated with improvements in key neonatal outcomes in this cohort. In contrast, infants delivered following spontaneous labor or medically indicated delivery demonstrated comparable, and in some cases more favorable, postnatal trajectories. These findings question the clinical utility of bowel‐related concerns, as documented in clinical practice, as a sole indication for delivery in gastroschisis and support reliance on established obstetric surveillance and guideline‐consistent decision‐making when determining delivery timing, within the context of the study's limitations.

## 1 INTRODUCTION

Gastroschisis is a condition with a prevalence of 1 in every 3268 live births [1]. The etiology of gastroschisis is still unknown, and no consensus exists for the initial neonatal management [2, 3]. Gastroschisis causes a disproportionate amount of resource use in comparison with other Neonatal Intensive Care Unit (NICU) admission conditions [4]. Many systematic reviews and retrospective studies recommend a high‐quality Randomized Controlled Trial (RCT) design to identify an appropriate evidence‐based approach [3, 5]. This approach led to the GOOD study that examined the hypothesis that late preterm delivery at 35–35^+6^ weeks can be a superior option to term delivery at 38^+0–38+6^ weeks [6]. Various new studies revealed that this hypothesis is controversial due to the side effects of preterm birth, such as respiratory and feeding failure, length of hospital stay, and infections [7, 8, 9, 10, 11].

In contemporary practice, there is substantial variation in the timing of delivery for fetuses with gastroschisis, with ongoing debate regarding the balance between risks of prematurity and prenatal bowel compromise [3, 5]. This gestational window reflects a balance between competing clinical concerns: the potential risks of prolonged in utero exposure to amniotic fluid on the exteriorized bowel [12, 13] versus the well‐established neonatal morbidities associated with earlier delivery [14]. Prior studies examining delivery timing in gastroschisis have yielded conflicting results, with some suggesting potential benefits of earlier delivery, including reduced sepsis or earlier initiation of enteral feeding [15, 16], while others demonstrate increased risks related to prematurity, such as feeding intolerance, infection, and prolonged hospitalization [3, 10, 17].

Importantly, decisions regarding delivery timing in this gestational age range are often driven not solely by gestational age but also by the clinical indication prompting delivery, consistent with American College of Obstetricians and Gynecologists (ACOG) guidance distinguishing medically indicated from spontaneous deliveries before 39 weeks’ gestation [18]. In pregnancies complicated by gastroschisis, abnormal prenatal bowel findings, such as bowel dilation, wall thickening, or increased echogenicity, are commonly cited as justification for earlier delivery, despite limited evidence that bowel appearance alone predicts improved neonatal outcomes [3, 19].

This study examined neonatal outcomes according to indication for delivery among pregnancies with prenatally diagnosed gastroschisis delivered late preterm and early term. We hypothesized that delivery prompted by fetal bowel concern would not be associated with improved postnatal outcomes compared with delivery for medically indicated obstetric reasons or following spontaneous labor. By focusing on the indication for delivery within a clinically relevant gestational window, this study aims to better inform evidence‐based delivery decision‐making in pregnancies complicated by gastroschisis.

## 2 METHODS

### 2.1 Study design and population

This Institutional Review Board (IRB)‐approved, single‐center retrospective cohort study included pregnancies with a prenatal ultrasound diagnosis of gastroschisis and neonatal care at Boston Children's Hospital (BCH), delivered between 34 0⁄7 and 38 6⁄7 weeks of gestation from January 2000 through December 2023. As a tertiary referral center without an in‐house obstetric delivery unit, many patients were managed and delivered at outside institutions, with subsequent neonatal transfer. Therefore, delivery timing and indications reflect practices across referring providers rather than a single standardized institutional protocol. A total of 135 pregnancies with gastroschisis were identified through institutional obstetric and surgical databases. Among 116 neonates delivered between 34 0⁄7 and 38 6⁄7 weeks’ gestation, 83 had a documented reason for delivery and comprised the analytic cohort; 33 were excluded due to missing or indeterminate documentation of delivery indication. The final 83 neonates had a clearly documented indication for delivery and postnatal follow‐up at BCH comprised the final analytic cohort. This study was approved by the BCH IRB with a waiver of informed consent.

### 2.2 Inclusion and exclusion criteria

Inclusion criteria were as follows: (1) confirmed diagnosis of gastroschisis at birth; (2) availability of postnatal clinical outcomes at the study institution to ensure adequate data completeness; and (3) delivery within the study gestational age range with a documented reason for delivery. Exclusion criteria included lack of postnatal confirmation of gastroschisis or delivery outside the defined range.

### 2.3 Classification of delivery indication

Indications for delivery were abstracted from obstetric documentation and categorized into three mutually exclusive groups:

1. *Medically indicated delivery*: delivery prompted by maternal, fetal, or placental conditions, including but not limited to abnormal fetal surveillance findings such as non‐reassuring non‐stress testing or biophysical profile, and not attributed to bowel‐specific ultrasound findings.
2. *Spontaneous labor*: spontaneous onset of birth or preterm premature rupture of membranes without preceding fetal surveillance abnormalities or bowel‐related concerns.
3. *Fetal bowel concern*: delivery prompted by prenatal ultrasound findings concerning for bowel compromise, including persistent bowel dilation, bowel wall thickening, increased echogenicity, or intra‐abdominal bowel dilation. Sonographic findings were not independently re‐reviewed or standardized for this study; classification was based on documentation in the medical record indicating that delivery was prompted by concern for bowel‐related abnormalities on prenatal ultrasound.

In cases where bowel‐related ultrasound findings coexisted with abnormal fetal surveillance or other obstetric indications, deliveries were classified as medically indicated; the fetal bowel concern group was limited to cases without concurrent obstetric indications.

### 2.4 Data collection and outcomes

Data were collected via standardized retrospective chart review by two independent reviewers, with discrepancies resolved by consensus. Collected variables included gestational age at delivery, delivery indication, mode of delivery, birth characteristics, and postnatal clinical outcomes. Detailed indications for cesarean delivery were not consistently available and were therefore not analyzed.

Primary neonatal outcomes were age at abdominal wall closure and length of hospital stay. Secondary outcomes included time to initiation of enteral feeding, number of surgical procedures in the first month of life, number of surgical procedures in the first year of life, incidence of neonatal sepsis, Silo reduction, and rate of complex gastroschisis. Neonatal sepsis was defined as culture‐proven infection requiring antibiotic treatment. Complex gastroschisis was defined based on postnatal findings, including operative and clinical documentation of bowel complications such as atresia, perforation, necrosis, or volvulus. Additional neonatal outcomes, including necrotizing enterocolitis and respiratory complications, were reviewed but not included in the primary analysis due to low event frequency.

### 2.5 Statistical analysis

Continuous variables are reported as mean ± standard deviation and categorical variables as frequencies and percentages. No a priori sample size or power calculation was performed given the retrospective design, and subgroup sizes may limit the ability to detect small differences. Given non‐normal distributions and small group sizes, comparisons across delivery indication groups were performed using the Kruskal–Wallis test for continuous variables and Fisher's exact test for categorical variables. Gestational age at delivery was prespecified as a key confounder, and adjusted analyses were performed using generalized linear models or ANCOVA, as appropriate. Adjusted analyses were performed using regression models with gestational age at delivery included as a covariate. Analyses were conducted using a complete‐case approach; denominators, therefore, varied by outcome. All tests were two‐sided, with *p* < 0.05 considered statistically significant.

## 3 RESULTS

### 3.1 Study population and delivery indication

This study was approved by the IRB with a waiver of informed consent. Among 135 fetuses with prenatally diagnosed gastroschisis identified during the study period, 116 neonates were delivered within the prespecified gestational age window for analysis (34 0⁄7–38 6⁄7 weeks’ gestation). Of these, 83 neonates had a clearly documented delivery classification and comprised the analytic cohort; 33 were excluded due to missing or indeterminate documentation precluding classification.

Among the 83 included neonates, 36 (43.4%) were delivered for medically indicated delivery, 22 (26.5%) were delivered for fetal bowel concern based on prenatal ultrasound findings, and 25 (30.1%) were delivered following spontaneous labor. Gestational age at delivery was similar across delivery classification groups. Baseline neonatal characteristics, including gestational age at delivery, birth weight, sex, and mode of delivery, were comparable across groups (Table 1). There were no fetal demises within the studied gestational age range. There was one neonatal death within 30 days of birth in the overall cohort, which occurred in a case without a documented indication for delivery. The death was attributed to complications of severe bowel disease, including necrotic intestinal involvement, associated with respiratory acidosis and hemodynamic instability. The proportion of neonates with complex gastroschisis did not differ significantly across delivery classification groups in either unadjusted or adjusted analyses (Table 2).

**TABLE 1:** Baseline maternal and neonatal characteristics by reason for delivery in gastroschisis.

Characteristic	Fetal bowel Concern (*n* = 22)	Medically indicated delivery (*n* = 36)	Spontaneous labor (*n* = 25)	Not assigned (33)
Gestational age at delivery, weeks	35.83 ± 1.31	35.85 ± 1.05	35.93 ± 1.17	36.65 ± 1.07
Gestational age at diagnosis, weeks	16.8 ± 3.2	15.5 ± 3.0	17.6 ± 3.2	18.01 ± 4.38
Birth weight, g	2374 ± 352	2255 ± 350	2359 ± 373	2581.85 ± 514.97
Male sex, %	10/22 (45.5)	15/36 (41.7)	18/25 (72.0)	19/33 (57)
Cesarean delivery, *n*/*N* (%)	15/22 (68.2)	27/36 (75.0)	10/24 (41.7)	18/32 (56.2)

*Note*: Values are mean ± SD or *n* (%). Percentages are calculated using available denominators.

**TABLE 2:** Neonatal outcomes by reason for delivery in prenatally diagnosed gastroschisis (34^0^–38^6^ weeks’ gestation).

Outcome	Fetal bowel concern (*n* = 22)	Medically indicated delivery (*n* = 36)	Spontaneous labor (*n* = 25)	Unadjusted *p* ^†^	Adjusted *p* (vs. fetal bowel concern)
Age at abdominal closure (days)	6 (1–170)	4 (1–13)	4 (1–12)	0.08	**0.013 (M)** **0.025 (S)**
Surgeries in the first month	2 (1–5)	2 (1–3)	2 (1–5)	0.51	0.186 (M) 0.415 (S)
Surgeries in the first year	3 (1–9)	2 (1–6)	2 (1–4)	0.63	0.352 (M) 0.237 (S)
Length of stay (LOS) (days)	55 (27–360)	46.5 (14–187)	42 (17–171)	0.32	0.269 (M) 0.573 (S)
Sepsis rate	18.2%	14.3%	20.0%	0.81	0.700 (M) 0.855 (S)
Age at enteral feeding (days)	35 (12–3285)	25 (9–3285)	31.5 (15–145)	0.21	0.750 (M) 0.659 (S)
Complex gastroschisis rate	45.0%	36.4%	54.2%	0.41	0.826 (M) 0.778 (S)
Silo reduction	81.8%	68.6%	76.0%	0.52	0.254 (M) 0.562 (S)

*Note*: Values are median (range) or *n* (%). Analyses were performed using a complete‐case approach; denominators varied by outcome based on data availability. Abbreviations: M, medically indicated delivery; S, spontaneous labor.

^†^ Kruskal–Wallis test used for continuous outcomes; Fisher's exact test used for categorical outcomes.

### 3.2 Feeding outcomes

Age at initiation of enteral feeding did not differ significantly by delivery classification. Mean age at initiation of enteral feeding was 44.9 ± 34.9 days among neonates delivered for fetal bowel concern, 36.9 ± 35.2 days among those delivered for medically indicated delivery, and 57.0 ± 42.7 days among those delivered following spontaneous labor (*p* = 0.21). After adjustment for gestational age at delivery, no statistically significant differences were observed between groups (Table 2).

### 3.3 Infectious outcomes

Rates of neonatal sepsis were similar across delivery classification groups. Sepsis occurred in 18.2% of neonates delivered for fetal bowel concern, 14.3% for medically indicated delivery, and 20.0% for spontaneous labor (*p* = 0.81). These findings remained non‐significant after adjustment for gestational age at delivery (Table 2).

### 3.4 Length of hospital stay

Length of hospital stay did not differ significantly by delivery classification. Mean length of stay was 82.6 ± 65.3 days for neonates delivered for fetal bowel concern, 60.2 ± 47.0 days for those delivered for medically indicated delivery, and 66.9 ± 46.4 days for those delivered following spontaneous labor (*p* = 0.32). Adjustment for gestational age at delivery did not materially alter these findings (Table 2).

### 3.5 Surgical outcomes

The number of surgical procedures performed during the first month of life was similar across delivery classification groups (bowel concern: 2.3 ± 1.2; medically indicated delivery: 1.9 ± 0.5; spontaneous labor: 2.0 ± 0.9; *p* = 0.51), with no significant differences observed after adjustment for gestational age at delivery. Similarly, the number of surgical procedures performed during the first year of life did not differ significantly among groups (Table 2). The proportion of neonates requiring silo placement did not differ significantly across delivery indication groups (81.8% vs. 68.6% vs. 76.0%, *p* = 0.52), and remained non‐significant after adjustment for gestational age at delivery.


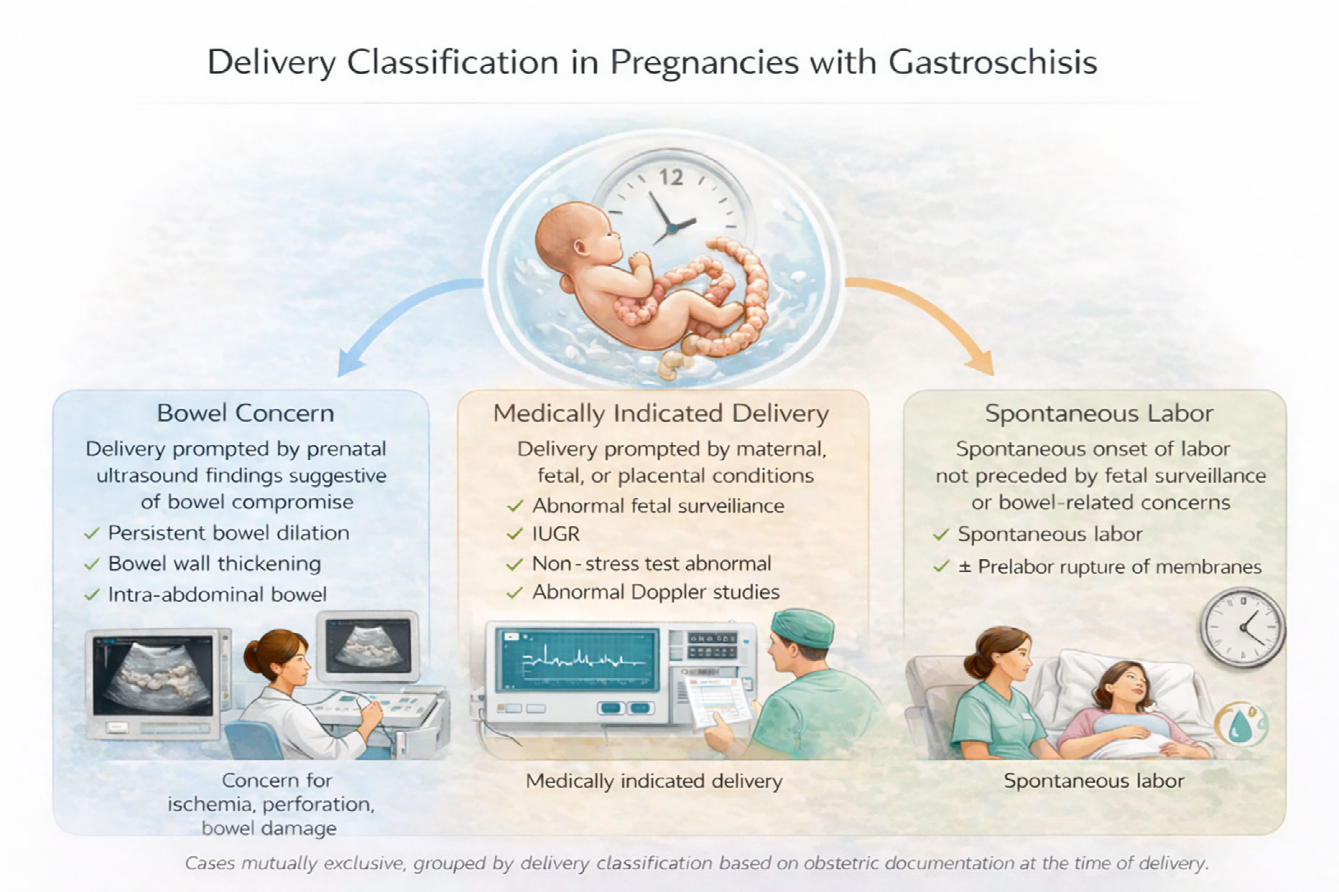


In contrast, age at abdominal wall closure differed by delivery classification. After adjustment for gestational age at delivery, neonates delivered for medically indicated delivery and spontaneous labor underwent abdominal wall closure at an earlier postnatal age compared with those delivered for fetal bowel concern (adjusted *p* = 0.013 and *p* = 0.025, respectively; Table 2).

Overall, delivery classification was not associated with differences in feeding outcomes, infectious complications, length of hospitalization, surgical burden, or disease complexity. Earlier abdominal wall closure was observed among neonates delivered for medically indicated delivery and spontaneous labor compared with fetal bowel concern, without corresponding differences in other clinically meaningful outcomes.

## 4 DISCUSSION

In this single‐center retrospective cohort of 83 pregnancies with prenatally diagnosed gastroschisis delivered between 34 0⁄7 and 38 6⁄7 weeks’ gestation, we compared neonatal outcomes according to delivery classification, including fetal bowel concern, medically indicated delivery, and spontaneous labor. No fetal demises were observed in this cohort, limiting assessment of the relationship between delivery timing and prevention of stillbirth. Across delivery classification groups, there were no significant differences in feeding outcomes, neonatal sepsis, length of hospitalization, surgical burden, or disease complexity. Age at abdominal wall closure was the only outcome that differed by delivery classification, occurring later among infants delivered for fetal bowel concern compared with those delivered following medically indicated delivery or spontaneous labor. Additionally, the proportion of neonates requiring silo placement was similar across groups, suggesting that differences in time to abdominal wall closure are unlikely to be explained solely by variation in initial closure approach. Although prolonged time to closure in individual cases may reflect anatomic complexity or intraoperative decision‐making, these factors were not systematically captured. In this cohort, delivery prompted by bowel‐related ultrasound findings was not associated with improvement in the measured postnatal outcomes. The proportion of complex gastroschisis in this cohort is higher than commonly reported, likely reflecting referral bias at a tertiary care center where more severe cases are preferentially managed.

In the general obstetric population, medically indicated delivery is often associated with higher neonatal morbidity than spontaneous birth, reflecting the underlying maternal or fetal pathology prompting early delivery [20]. In contrast, within our gastroschisis cohort, neonatal outcomes were similar between fetal bowel concern and the other delivery classification groups after accounting for gestational age at delivery. This pattern suggests that, in this cohort, earlier delivery driven by a perceived fetal “signal” does not necessarily confer benefit, particularly when that signal is based on bowel appearance rather than abnormal fetal surveillance. Gastroschisis exposes fetal bowel to amniotic fluid and is associated with inflammatory change that may worsen with ongoing gestation [21]. This mechanistic concern, together with reported stillbirth risk later in gestation [22], has driven ongoing debate regarding the timing of delivery. However, earlier delivery introduces well‐established related risks, including respiratory morbidity, feeding delay, infection, and longer hospitalization [23, 24, 25]. Consistent with this, multicenter data demonstrate that delivery before 35 weeks is associated with greater need for respiratory and feeding support and longer hospitalization compared with later gestational ages [7]. Several decision‐analytic studies have therefore suggested that delivery near 38 weeks may offer a pragmatic balance between prematurity risks and concerns related to prolonged intrauterine exposure [26, 27]. Importantly, our findings do not provide evidence to support deviation from standard obstetric timing solely on the basis of bowel appearance in the absence of abnormal fetal surveillance.

While various radiologic features have been linked to adverse neonatal outcomes, these findings support consideration of standard obstetric indications [18] when determining the timing of delivery. Several prenatal imaging findings have been identified as potential predictors of complications in gastroschisis, including intra‐abdominal bowel dilation (IABD), where an increased intra‐abdominal intestinal diameter (IAID) on prenatal ultrasound is associated with prolonged time to full enteral feeding and a higher likelihood of complex gastroschisis [28]; polyhydramnios, which has been strongly linked to severe bowel complications in the neonatal period [29]; and bowel wall thickening and edema [30], which are sonographic markers associated with adverse neonatal outcomes, including significant bowel injury. However, in our cohort, delivery prompted by these imaging findings, before any fetal distress, was not associated with improvement in postnatal clinical outcomes. Taken together, these findings suggest that bowel sonographic abnormalities may be useful for risk stratification and counseling, but they are insufficient as stand‐alone triggers for earlier delivery without corroborating fetal‐status concerns. Despite the associations between certain radiologic features and poor outcomes, current evidence does not support altering standard obstetric management solely based on these prenatal imaging findings [3, 19]. A randomized controlled trial comparing early delivery at 34 weeks to routine obstetric care found no benefit to early delivery; in fact, early delivery was associated with increased risks, such as late‐onset sepsis [11]. Our results reinforce previous studies [26] that bowel appearance alone should not supersede established obstetric surveillance in determining delivery timing for gastroschisis.

This retrospective study, spanning 20 years at a single referral center, provides insight into neonatal outcomes associated with different reasons for delivery in pregnancies complicated by gastroschisis. A key strength is the subgroup analysis distinguishing deliveries prompted by obstetric indications, bowel‐related concerns, and spontaneous labor, allowing for a more nuanced evaluation of clinical decision‐making. However, the study's modest sample size and division into multiple groups limit statistical power and generalizability. Indications for cesarean delivery were not consistently documented, limiting further interpretation of delivery mode. Some neonatal outcomes of interest, including necrotizing enterocolitis and respiratory complications, were too infrequent to allow meaningful comparison across groups. As a single‐center retrospective analysis, findings may reflect center‐specific practices and are subject to selection bias. Exclusion of cases with missing or indeterminate documentation of delivery indication may have introduced selection bias, and temporal changes in prenatal management, neonatal care (including TPN practices and infection management), and surgical approaches over the 20‐year study period may confound comparisons, although neonatal intensive care and surgical approaches for gastroschisis were generally stable at our institution over the study period. Because inclusion required a clearly documented clinical indication for delivery, cases without this information (33 of 116 cases, 28%) could not be reliably classified. Although exclusion was based on documentation availability rather than clinical outcomes, included cases may differ systematically from those not analyzed, potentially affecting generalizability. Residual confounding cannot be excluded. Additionally, sonographic indicators of bowel concern were not systematically re‐evaluated or standardized across the study period, and classification relied on clinical documentation. Variability in ultrasound technique, interpretation, and reporting over the 20‐year period may have influenced classification and represents a potential source of misclassification bias.

In summary, these data support a surveillance‐first approach to delivery timing in pregnancies complicated by gastroschisis that is consistent with current ACOG guidance for late‐preterm and early‐term delivery, while recognizing that prospective studies are needed to further define optimal delivery strategies. When bowel abnormalities are identified on prenatal ultrasound, management should prioritize intensified fetal surveillance and established obstetric indications—such as non‐reassuring fetal testing, growth restriction, or abnormal Doppler studies—rather than delivery based on bowel appearance alone. Adherence to this guideline‐consistent approach may avoid unnecessary earlier delivery without demonstrable improvement in postnatal outcomes.

## CONFLICT OF INTEREST STATEMENT

The authors declare no conflicts of interest.

## FUNDING INFORMATION

The authors received no specific funding for this work.

## Data Availability

The data that support the findings of this study are available from the corresponding author upon reasonable request.

## References

[1] Feldkamp, M. L. , M. A. Canfield , S. Krikov , D. Prieto‐Merino , A. Šípek , N. LeLong , E. Amar , et al. 2024. “Gastroschisis Prevalence Patterns in 27 Surveillance Programs From 24 Countries, International Clearinghouse for Birth Defects Surveillance and Research, 1980–2017.” Birth Defects Research 116(2): e2306. 10.1002/bdr2.2306 PMC11182352 38411327

[2] Stoll, C. , Y. Alembik , B. Dott , and M. P. Roth . 2008. “Omphalocele and Gastroschisis and Associated Malformations.” American Journal of Medical Genetics. Part A 146A(10): 1280–5. 10.1002/ajmg.a.32297 18386803

[3] Slidell, M. B. , J. McAteer , D. Miniati , S. Sømme , D. Wakeman , K. Rialon , D. Lucas , et al. 2024. “Management of Gastroschisis: Timing of Delivery, Antibiotic Usage, and Closure Considerations (A Systematic Review From the American Pediatric Surgical Association Outcomes & Evidence Based Practice Committee).” Journal of Pediatric Surgery 59(8): 1408–17. 10.1016/j.jpedsurg.2024.03.044 38796391

[4] Sydorak, R. M. , A. Nijagal , L. Sbragia , S. Hirose , K. Tsao , R. H. Phibbs , S. K. Schmitt , et al. 2002. “Gastroschisis: Small Hole, Big Cost.” Journal of Pediatric Surgery 37(12): 1669–72. 10.1053/jpsu.2002.36689 12483626

[5] Allin, B. S. , W. H. Tse , S. Marven , P. R. Johnson , and M. Knight . 2015. “Challenges of Improving the Evidence Base in Smaller Surgical Specialties, as Highlighted by a Systematic Review of Gastroschisis Management.” PLoS ONE 10(1): e0116908. 10.1371/journal.pone.0116908 PMC4306505 25621838

[6] Wagner, A. J. Gastroschisis Outcomes of Delivery (GOOD) Study. ClinicalTrialsgov. 2016.

[7] Riddle, S. , K. Acharya , N. Agarwal , I. Ahmad , E. Bendel‐Stenzel , J. Shepherd , S. Williams , I. Zaniletti , and E. Jacobson ; Children's Hospitals Neonatal Consortium's Gastroschisis Focus Group . 2024. “Gestational Age at Delivery and Neonatal Outcomes Among Infants With Gastroschisis in the Children's Hospitals Neonatal Consortium (CHNC).” American Journal of Perinatology 41(6): 756–63. 10.1055/s-0042-1744510 35553040

[8] Goldstein, M. J. , J. M. Bailer , and V. M. Gonzalez‐Brown . 2022. “Preterm vs Term Delivery in Antenatally Diagnosed Gastroschisis: A Systematic Review and Meta‐Analysis.” American Journal of Obstetrics & Gynecology MFM 4(4): 100651. 10.1016/j.ajogmf.2022.100651 35462060

[9] Tosello, B. , M. Zahed , F. Guimond , K. Baumstarck , A. Faure , F. Michel , O. Claris , et al. 2017. “Management and Outcome Challenges in Newborns With Gastroschisis: A 6‐Year Retrospective French Study.” Journal of Maternal‐Fetal & Neonatal Medicine 30(23): 2864–70. 10.1080/14767058.2016.1265935 27892784

[10] Carnaghan, H. , D. Baud , E. Lapidus‐Krol , G. Ryan , P. S. Shah , A. Pierro , and S. Eaton . 2016. “Effect of Gestational Age at Birth on Neonatal Outcomes in Gastroschisis.” Journal of Pediatric Surgery 51(5): 734–8. 10.1016/j.jpedsurg.2016.02.013 PMC4918692 26932253

[11] Shamshirsaz, A. A. , T. C. Lee , A. B. Hair , H. Erfani , J. Espinoza , A. A. Shamshirsaz , K. A. Fox , et al. 2020. “Elective Delivery at 34 Weeks vs Routine Obstetric Care in Fetal Gastroschisis: Randomized Controlled Trial.” Ultrasound in Obstetrics & Gynecology 55(1): 15–9. 10.1002/uog.21871 31503365

[12] Bhat, V. , M. Moront , and V. Bhandari . 2020. “Gastroschisis: A State‐of‐the‐Art Review.” Children 7(12): 302. 10.3390/children7120302 PMC7765881 33348575

[13] Langer, J. C. , J. G. Bell , R. O. Castillo , T. M. Crombleholme , M. T. Longaker , B. W. Duncan , S. M. Bradley , W. E. Finkbeiner , E. D. Verrier , and M. R. Harrison , 1990. “Etiology of Intestinal Damage in Gastroschisis, II. Timing and Reversibility of Histological Changes, Mucosal Function, and Contractility.” Journal of Pediatric Surgery 25(11): 1122–6. 10.1016/0022-3468(90)90745-u 2148773

[14] Sengupta, S. , V. Carrion , J. Shelton , R. J. Wynn , R. M. Ryan , K. Singhal , and S. Lakshminrusimha . 2013. “Adverse Neonatal Outcomes Associated With Early‐Term Birth.” JAMA Pediatrics 167(11): 1053–9. 10.1001/jamapediatrics.2013.2581 24080985

[15] Landisch, R. M. , Z. Yin , M. Christensen , A. Szabo , and A. J. Wagner . 2017. “Outcomes of Gastroschisis Early Delivery: A Systematic Review and Meta‐Analysis.” Journal of Pediatric Surgery 52(12): 1962–71. 10.1016/j.jpedsurg.2017.08.068 28947324

[16] Baud, D. , A. Lausman , M. A. Alfaraj , G. Seaward , J. Kingdom , R. Windrim , J. C. Langer , E. N. Kelly , and G. Ryan , 2013. “Expectant Management Compared With Elective Delivery at 37 Weeks for Gastroschisis.” Obstetrics and Gynecology 121(5): 990–8. 10.1097/AOG.0b013e31828ec299 23635735

[17] Overcash, R. T. , D. A. DeUgarte , M. L. Stephenson , R. M. Gutkin , M. E. Norton , S. Parmar , M. Porto , F. R. Poulain , and D. B. Schrimmer , 2014. “Factors Associated With Gastroschisis Outcomes.” Obstetrics and Gynecology 124(3): 551–7. 10.1097/AOG.0000000000000425 PMC4147679 25162255

[18] 2021. “Medically Indicated Late‐Preterm and Early‐Term Deliveries: ACOG Committee Opinion, Number 831.” Obstetrics and Gynecology 138(1): e35–e9. 10.1097/AOG.0000000000004447 34259491

[19] Oakes, M. C. , M. Porto , and J. H. Chung . 2018. “Advances in Prenatal and Perinatal Diagnosis and Management of Gastroschisis.” Seminars in Pediatric Surgery 27(5): 289–99. 10.1053/j.sempedsurg.2018.08.006 30413259

[20] Wing, D. 2016. “412: Comparison of Neonatal Outcomes Among Indicated and Spontaneous Preterm Births From the nuMoM2b Study.” American Journal of Obstetrics & Gynecology 214(1): S226–S7.

[21] Frascoli, M. , C. Jeanty , S. Fleck , P. W. Moradi , S. Keating , A. N. Mattis , Q. Tang , and T. C. MacKenzie , 2016. “Heightened Immune Activation in Fetuses With Gastroschisis May Be Blocked by Targeting IL‐5.” Journal of Immunology 196(12): 4957–66. 10.4049/jimmunol.1502587 PMC4977025 27183609

[22] Amin, R. , A. Domack , J. Bartoletti , E. Peterson , B. Rink , J. Bruggink , M. Christensen , A. Johnson , W. Polzin , and A. J. Wagner , 2019. “National Practice Patterns for Prenatal Monitoring in Gastroschisis: Gastroschisis Outcomes of Delivery (GOOD) Provider Survey.” Fetal Diagnosis and Therapy 45(2): 125–30. 10.1159/000487541 29791899

[23] Jaczyńska, R. , D. Mydlak , B. Mikulska , A. Nimer , T. Maciejewski , and E. Sawicka . 2023. “Perinatal Outcomes of Neonates With Complex and Simple Gastroschisis After Planned Preterm Delivery—A Single‐Centre Retrospective Cohort Study.” Diagnostics 13(13): 2225. 10.3390/diagnostics13132225 PMC10340363 37443619

[24] Boyle, E. M. , F. J. Mielewczyk , and C. Mulvaney . 2024. “Late Preterm and Early Term Birth: Challenges and Dilemmas in Clinical Practice.” Seminars in Fetal and Neonatal Medicine 29(6): 101564. 10.1016/j.siny.2024.101564 39523171

[25] Boyle, E. M. , S. Johnson , B. Manktelow , S. E. Seaton , E. S. Draper , L. K. Smith , J. Dorling , N. Marlow , S. Petrou , and D. J. Field , 2015. “Neonatal Outcomes and Delivery of Care for Infants Born Late Preterm or Moderately Preterm: A Prospective Population‐Based Study.” Archives of Disease in Childhood—Fetal and Neonatal Edition 100(6): F479–F85. 10.1136/archdischild-2014-307347 PMC4680176 25834169

[26] Harper, L. M. , K. R. Goetzinger , J. R. Biggio , and G. A. Macones . 2015. “Timing of Elective Delivery in Gastroschisis: A Decision and Cost‐Effectiveness Analysis.” Ultrasound in Obstetrics & Gynecology 46(2): 227–32. 10.1002/uog.14721 PMC4861040 25377308

[27] Packer, C. H. , R. A. Pilliod , A. B. Caughey , and T. N. Sparks . 2023. “Optimal Timing of Delivery for Growth Restricted Fetuses With Gastroschisis: A Decision Analysis.” Prenatal Diagnosis 43(12): 1506–13. 10.1002/pd.6452 37853803

[28] Dewberry, L. C. , S. A. Hilton , M. V. Zaretsky , N. Behrendt , H. L. Galan , A. I. Marwan , and K. W. Liechty . 2020. “Examination of Prenatal Sonographic Findings: Intra‐Abdominal Bowel Dilation Predicts Poor Gastroschisis Outcomes.” Fetal Diagnosis and Therapy 47(3): 245–50. 10.1159/000501592 31454815

[29] Japaraj, R. P. , R. Hockey , and F. Y. Chan . 2003. “Gastroschisis: Can Prenatal Sonography Predict Neonatal Outcome?.” Ultrasound in Obstetrics & Gynecology 21(4): 329–33. 10.1002/uog.85 12704738

[30] Sinkey, R. G. , M. A. Habli , A. P. South , W. W. Gibler , P. W. Burns , M. A. Eschenbacher , and C. R. Warshak . 2016. “Sonographic Markers Associated With Adverse Neonatal Outcomes Among Fetuses With Gastroschisis: An 11‐Year, Single‐Center Review.” American Journal of Obstetrics & Gynecology 214(2): 275.e1–e7. 10.1016/j.ajog.2015.09.081 26454131

